# Highly enhanced optical properties of indocyanine green/perfluorocarbon nanoemulsions for efficient lymph node mapping using near-infrared and magnetic resonance imaging

**DOI:** 10.1186/s40580-014-0006-6

**Published:** 2014-03-14

**Authors:** Pan Kee Bae, Juyeon Jung, Bong Hyun Chung

**Affiliations:** Bionanotechnology Research Center, Korea Research Institute of Bioscience and Biotechnology, Daejeon, 305-806 South Korea

**Keywords:** NIR optical imaging, Indocyanine green, bimodal imaging, ^19^ F-MR imaging, Lymph node mapping

## Abstract

The near-infrared (NIR) fluorescence probe has better tissue penetration and lower autofluorescence. Indocyanine green (ICG) is an NIR organic dye for extensive biological application, and it has been clinically approved for human medical imaging and diagnosis. However, application of this dye is limited by its numerous disadvantageous properties in aqueous solution, including its concentration-dependent aggregation, poor aqueous stability in vitro, and low quantum yield. Its use in molecular imaging probes is limited because it loses fluorescence after binding to nonspecific plasma proteins, leading to rapid elimination from the body with a half-life of 2 – 4 min. In this study, the multifunctional perfluorocarbon (PFC)/ICG nanoemulsions were investigated with the aim of overcoming these limitations. The PFC/ICG nanoemulsions as a new type of delivery vehicle for contrast agents have both NIR optical imaging and ^19^ F-MR imaging moieties. These nanoemulsions exhibited less aggregation, increased fluorescence intensity, long-term stability, and physicochemical stability against external light and temperature compared to free aqueous ICG. Also, the PFC/ICG bimodal nanoemulsions allow excellent detection of lymph nodes in vivo through NIR optical imaging and ^19^ F-MR imaging. This result showed the suitability of the proposed nanoemulsions for non-invasive lymph node mapping as they enable long-time detection of lymph nodes.

**Electronic supplementary material:**

The online version of this article (doi:10.1186/s40580-014-0006-6) contains supplementary material, which is available to authorized users.

## Background

Medical imaging has experienced explosive growth over the past few decades and now plays a central role in noninvasive imaging technologies for biomedical research. Multimodal imaging will allow clinicians not only to see where a tumor is located in the body but also to visualize the expression and activity of specific molecules and biological processes that influence tumor behaviour and/or its response to therapy. The multimodal imaging probes with optical imaging dyes and magnetic resonance (MR) imaging contrast agents have been exploited for targeted molecular imaging, disease diagnosis, and in vivo animal studies [1,2]. Targeted magnetic resonance imaging (MRI) has emerged as a promising diagnostic approach that offers a high-resolution depiction of pathological anatomy and the ability to detect associated disease biomarkers [3,4]. Fluorophores have long been used as luminescence probes in various biological and biomedical applications [5–7].

Perfluorocarbons (PFCs) have been widely employed as ^19^ F-MR agents for imaging modalities due to their biological and chemical inertness. PFCs have been used clinically as blood substitutes because of their high gas dissolving capacity for oxygen and carbon-dioxide as well as their chemical and metabolic stability [8]. The ^19^ F in the PFC nanoparticles have low background biological abundance and provide excellent signal sensitivity compared with ^1^H [9–11]. The encapsulation or conjugation of a wide variety of contrast agents onto PFCs for multimodal imaging and therapeutics, in combination with antibodies or other targeting ligands, causes them to accumulate in specific sites, holding great potential for medical applications. The PFC nanoemulsions have been widely explored for various applications, such as ultrasound imaging as a vehicle for targeted delivery of contranst agents and drug delivery [12], partial liquid lung ventilation [13], and blood substitutes [8,14].

Indocyanine green (ICG) is a water-soluble tricarbocyanine dye with substantial absorption and fluorescence in the near-infrared region (NIR) [15,16]. The United States Food and Drug Administration (FDA) [17] has approved ICG for use in diagnostic applications in clinical settings including its use as an optical contrast agent in the imaging of cardiac and hepatic vascular systems [18,19], the retinal and choroidal vasculature [20], and lymphatic systems [21–23]. ICG has also been investigated in laser-mediated therapeutic applications. This dye can transform from the absorbed NIR light energy into free-oxygen species and heat, which further expands its therapeutic application to photothermal and photodynamic therapies [24–26]. Moreover, ICG, a NIR fluorescence probe, has several advantages over visible optical probes for in vivo imaging applications, including improved deep-tissue penetration, lower absorption and scattering by blood and tissue components, and minimal autofluorescence [18,27]. However, ICG has several intrinsic limitations in optical imaging applications: 1) ICG is rapidly cleared from the circulatory system (half-life of 2-4 min) [28,29]; 2) ICG forms aggregates depending on its concentration and interacts non-covalently with various proteins such as lipoproteins, plasma proteins, and human serum albumin via physical mechanisms due to its amphiphilic properties [30,31]; 3) in aqueous solutions, ICG is unstable as the compound undergoes physicochemical transformations such as thermal degradation and photodegradation [28,32]. Such changes result in discoloration, decreased light absorption, decreased fluorescence, and a shift in the wavelength of maximum absorption.

Recently, researchers have proposed nanomaterial-based ICG probes to overcome the high degradation rate and the short plasma half-life of ICG [33–35]. Polymeric nanoparticles and inorganic nanoparticles containing ICG could increase the stability of ICG and improve its physicochemical stability [34,35]. Also, nanoparticles with a polymer core and lipid shell provided great targeting capability [36]. Liposomes have been widely used as fluorescence probes for in vivo applications. In this study, we synthesized multifunctional PFC/ICG nanoemulsions as a new type of delivery vehicles for ICG to overcome the aforementioned limitations. The PFC/ICG nanoemulsions have both NIR optical imaging and ^19^ F-MR imaging moieties. We demonstrated the stability and fluorescence intensity of PFC/ICG nanoemulsions in vivo and in vitro and their physicochemical stability against exterior light and temperature. Also, we used multi-modal PFC/ICG nanoemulsions to indentify sentinel lymph nodes.

## Methods

### Materials

Perfluoro-15-crown ether (PFCE) was obtained from SynQuest Laboratories Inc. (Alachua, FL). L-a-phosphatidylcholine (Egg PC), and 1,2-distearoyl-*sn*-glycero-3-phosphoethanolamine-*N*-[methoxy(polyethylene glycol)2000] (DSPE-mPEG_2000_) were purchased from Avanti Polar Lipids Inc. (Alabaster, AL). Indocyanine green, and cholesterol were obtained from Sigma-Aldrich Co. (St. Louis, MO).

### Preparation of PFC/ICG nanoemulsions

To synthesize the PFC/ICG nanoemulsions, PFCE liquids were emulsified in an aqueous solution using a lipid mixture. The lipid compositions of the PFC/ICG nanoemulsions were PC/cholesterol/DSPE-mPEG_2000_ in a molar ratio of 70:20:10, respectively. The lipid mixture was reacted for 1 h at room temperature by the addition of 2 mg ICG, evaporated with a rotary evaporator to ensure the production of a thin lipid film, and dried in a vacuum oven (25°C) for 24 h. The lipid film was rehydrated with phosphate-buffered saline (PBS), and the resulting solution was sonicated in a bath sonicator followed by five cycles of freezing and thawing. The rehydrated lipid mixture (2% w/v) and PFCE solution (20% v/v) were mixed for 4 min using a homogenizer, followed by microfluidisation [37]. A M-110S microfluidiser (Microfluidics Inc., Newton, MA) operating at a liquid pressure of approximately 20,000 psi was used for nanoemulsion preparations. The PFC/ICG nanoemulsions were stored at 4°C.

### Characterization of PFC/ICG nanoemulsions

To evaluate the characteristics of the PFC/ICG nanoemulsions, a JEOL FE-TEM (transmission electron microscope) was utilized, and the TEM images were captured at 200 kV using a device from Tecnai. The PFC/ICG nanoemulsions were drop-cast onto carbon-coated TEM grids preliminarily stained with 2% uranyl acetate, and the solution was dried in a vacuum oven.

The emission and absorption spectra were obtained on a Perkin-Elmer LS-55 and a Beckman Coulter UV–VIS spectrophotometer (DU 800). The size of the PFC/ICG nanoemulsions was analyzed via dynamic light scattering using an electrophoretic light scattering photometer (ELS-Z, Otsuka Electronics, Osaka, Japan). The NIR fluorescence images of the PFC/ICG nanoemulsions were obtained using the IVIS Lumina imaging system (Caliper Life Science, MA) with an ICG filter set.

### ICG and PFC loading efficiency

The ICG loading efficiency was analyzed using a previously reported method [29]. The quantity of ICG loaded into the PFC/ICG nanoemulsions was determined from the free ICG that was not incorporated into the PFC/ICG nanoemulsions. A 1-mL sample of the PFC/ICG nanoemulsion was centrifuged, and the supernatant was removed and stored in a centrifuge tube; the PFC/ICG nanoemulsion was dispersed in a PBS solution. The centrifugation was repeated, and the collected supernatants were combined. The ICG concentration was quantified via UV–Vis spectroscopy. The quantity of ICG inside the PFC/ICG nanoemulsions was also measured to verify the accuracy of the method. Selected PFC/ICG nanoemulsion samples were treated with an HNO_3_ solution to induce capsule disassembly and to release the ICG into the solution. For all tested samples, the quantity of ICG released and the unencapsulated ICG equaled the quantity of the ICG precursor, indicating that the mass balance was conserved. The loading efficiency was calculated as the mass of ICG incorporated by the PFC/ICG nanoemulsions divided by the total ICG mass added to the nanoemulsion aggregate suspension.

To evaluate the PFC loading efficiency in PFC/ICG nanoemulsions, 19 F-MR imaging was performed on serial dilutions (0 – 0.4 ml) of PFCE liquids using a 4.7 T Bruker scanner (Biospec, Rheinstetten, Germany). The 19 F MR signal intensity was determined from the PFC signals originating from the PFCE liquids within a region of interest (ROI). We generated a calibration curve from the serial dilutions of the PFCE liquids and calculated the PFC loading efficiency in PFC/ICG nanoemulssions. The loading efficiency of PFC onto the PFC/ICG nanoemulsions was approximately 75.5 ± 3.2%.

### Physicochemical stability of PFC/ICG nanoemulsions

The ICG and PFC/ICG nanoemulsions were diluted with distilled water to a final concentration of 1 μg/ml and were loaded onto a 12-well plate. The samples were irradiated with 760 nm NIR light from an LED for a predetermined time of 10, 20, 30, 60, or 120 min at room temperature. The effect of the light exposure on the degradation of the PFC/ICG nanoemulsions was determined with visible light at room temperature. The fluorescence intensity was measured for up to 6 days. After incubation, the remaining fluorescence of each sample was measured using a spectrofluorometer with excitation and emission wavelengths of 760 nm and 820 nm, respectively. For the quantitative analysis, we normalized the fluorescence signal intensity. This processing normalized the signal data points to the range [0, 1].

$$ Y'=\left(Y\hbox{--} {Y}_{\min}\right)/\left({Y}_{\max}\hbox{--} {Y}_{\min}\right) $$

*Y* denotes the y values of input curve, and *Y*’ is the normalized curve.

### Cell culture

The HeLa (human cervical cancer cells) and Raw264.7 (Murine macrophage cells) cell lines were obtained from the American Type Culture Collection (Rockville, MD). These cell lines were grown and maintained in Dulbecco’s modified Eagle’s medium (DMEM; Gibco BRL, Grand Island, NY) supplemented with 10% heat-inactivated fetal bovine serum (FBS), 50 IU/ml penicillin, and 50 μg/ml streptomycin. The cultures were maintained at 37°C/5% CO_2_ in tissue culture plates. The DC2.4 cells, previously characterized as an immature murine dendritic cell line, were obtained from Dr. Kenneth L. Rock (Dana-Farber Cancer Institute, Boston, MA) [38]. This cell line was grown and maintained in DMEM supplemented with 10% heat-inactivated FBS, 50 IU/ml penicillin, and 50 μg/ml streptomycin.

### Cell fluorescence imaging

To determine the intracellular delivery capacity of PFC/ICG nanoemulsions, the HeLa, Raw264.7, and DC2.4 cells were incubated with 10 μl/ml PFC/ICG nanoemulsions in μ-slide 8-well microscopy chamber at a density of 1 × 10^4^ cells per well for 6 h at 37°C. The culture medium was then carefully aspirated, and the cells were washed three times. The labeled cells were fixed with 4% paraformaldehyde and stained with DAPI. The NIR fluorescence images were obtained on a Deltavision RT deconvolution microscope (Applied Precision Technologies, Issaquah, WA) using a filter set (excitation: 775/50, emission: 845/55; Omega Optical, Brattleboro, VT).

### In vitro ^19^ F-MR and NIR fluorescence imaging

HeLa, Raw, or DC2.4 cells (1 × 10^6^) were seeded on each well of a 6-well plate and grown for 24 h. The cells were then incubated with a medium containing 10 μl/ml PFC/ICG nanoemulsions. After 6 h, the medium was removed, and the cells were washed three times with PBS. The cell pellets were suspended with a 2% solution of low-melting agarose. The cells were collected in 0.2-mL tubes, and the MR and NIR fluorescence signals were measured. All ^19^ F-MR imaging of the PFC/ICG nanoemulsions was performed with a 4.7 T Bruker scanner using a double-tuned ^1^H/^19^F quadrature birdcage RF resonator. The ^19^ F-MR image was captured with a FLASH sequence (128 × 128 matrix; 30 × 30 mm^2^ FOV; 50 ms TR; 2.6 ms TE; 10 mm slice thickness; 256 NEX). The NIR fluorescence images were obtained using the IVIS Lumina imaging system (Caliper Life Science, MA) with an ICG filter set.

### Cell cytotoxicity assays

The cell cytotoxicity was assessed using a modified 3-(4, 5-dimethylthiazol-2-yl)-2, 5-diphenyltetrazolium bromide (MTT) assay. Raw, HeLa, or DC2.4 cells were seeded in a 96-well plate (Corning Costar, Cambridge, MA) at 1 × 10^4^ cells/well. After incubation for 24 or 48 h, several different concentrations of the prepared PFC/ICG nanoemulsions (0.38 ug/ul of ICG, 0.3 ul/ul of PFC) were poured into the wells. After incubation for a predetermined time, the residual nanoemulsions were removed, and a 2.5 mg/ml MTT solution was added to each well. The wells were then incubated in a humidified CO_2_ incubator at 37°C for 2 h. An acidified isopropanol/10% Triton X-100 solution (100 μl) was then added, and the plates were shaken to dissolve the formazan products. The absorbance was measured using a microplate reader at 570 nm. The cell survival rate in the control wells without the PFC/ICG nanoemulsions was considered 100% cell survival. The cytotoxic concentration (CC_80_) was defined as the concentration of the compound that reduced the absorbance of the control samples by 80%.

### In vivo tracking of PFC/ICG nanoemulsions using NIR fluorescence and ^19^ F-MR imaging

Female hairless mice, 5–6 weeks of age, were purchased from SLC, Inc. (Japan). The mice were maintained at the KRIBB animal facility under pathogen-free conditions. All animal care and experimental procedures were approved by the Animal Care Committees of the KRIBB.

For the in vivo NIR fluorescence and ^19^ F-MR imaging of the sentinel lymph nodes, hairless mice were injected with 20 μl (25 μM of ICG, 15 ul/ml of PFC) of the PFC/ICG nanoemulsions (n = 5) or free ICG solutions (n = 5) in the footpad of the foreleg. Prior to the fluorescence imaging experiments, the mice were anesthetized with 200 μl of a 2.5% avertin solution (2, 2, 2-tribromoethanol-tert amyl alcohol, Sigma) throughout the experiments. After a predetermined time, the fluorescence intensity was quantitatively analyzed using the IVIS Lumina imaging system. Thereafter, the ^19^ F-MR images of the mice were obtained with a 4.7 T Bruker scanner using a double-tuned ^1^H/^19^F Birdcage coil design (inner diameter: 35 mm; length: 78 mm). After acquiring the morphological ^1^H images, the resonator was tuned to ^19^ F. For the ^19^ F-MR image, the mouse was imaged with a gradient echo sequence (128 × 128 matrix; 3 cm FOV; 56.0 ms TR; 2.6 ms TE; 20 mm slice thickness; 60° flip angle; 256 NEX; 30 min total scan time).

### Statistical analysis

The statistical evaluations of the experiments were performed by ANOVA analysis followed by a Newman-Keuls multiple comparison test.

## Results and discussion

### Characteristics of PFC/ICG nanoemulsions

**Scheme 1:**
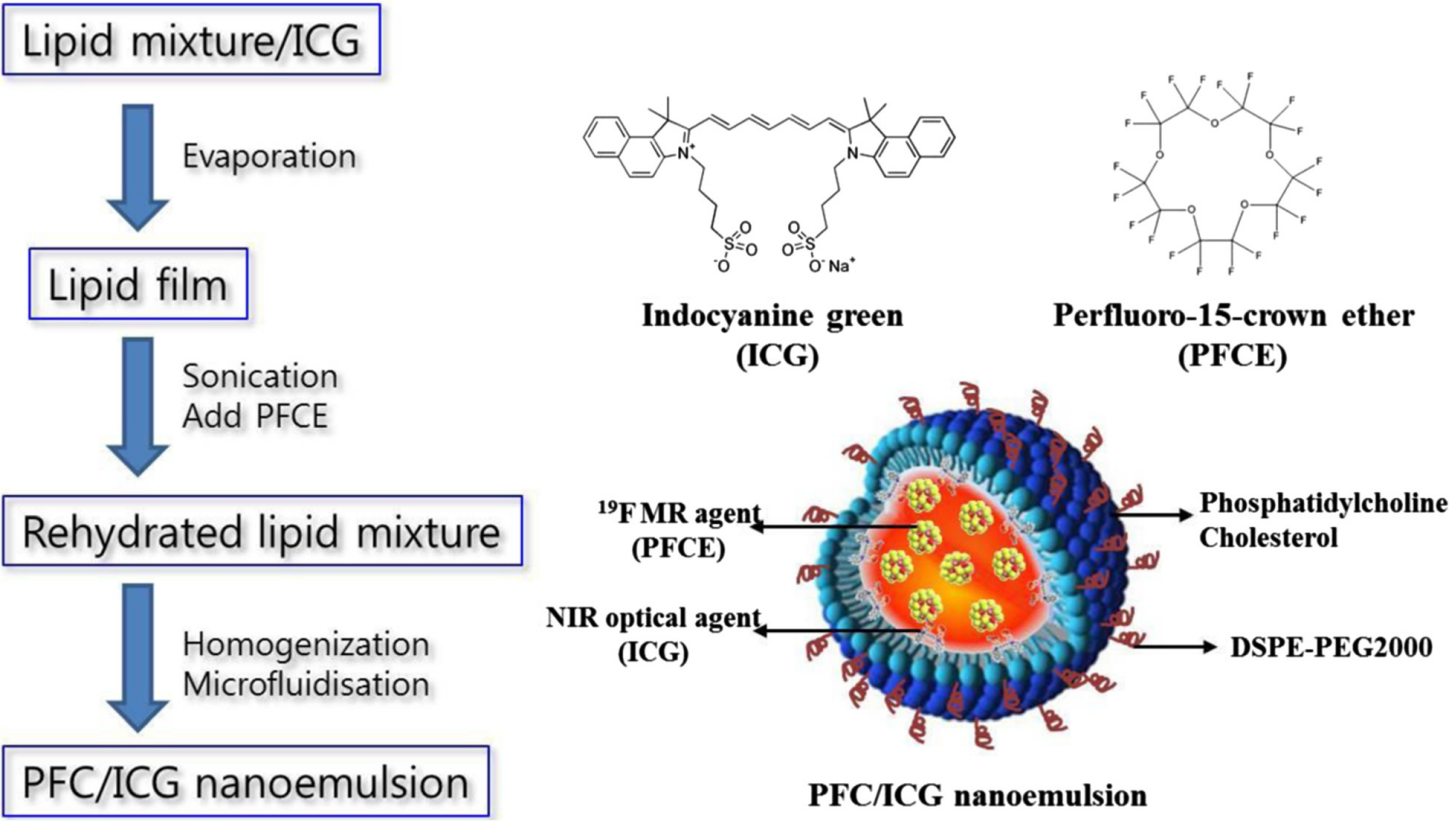
**Schematic illustration of the PFC/ICG nanoemulsions having both** ^**19**^ **F-MR and NIR optical imaging capabilities.**

**Figure 1:**
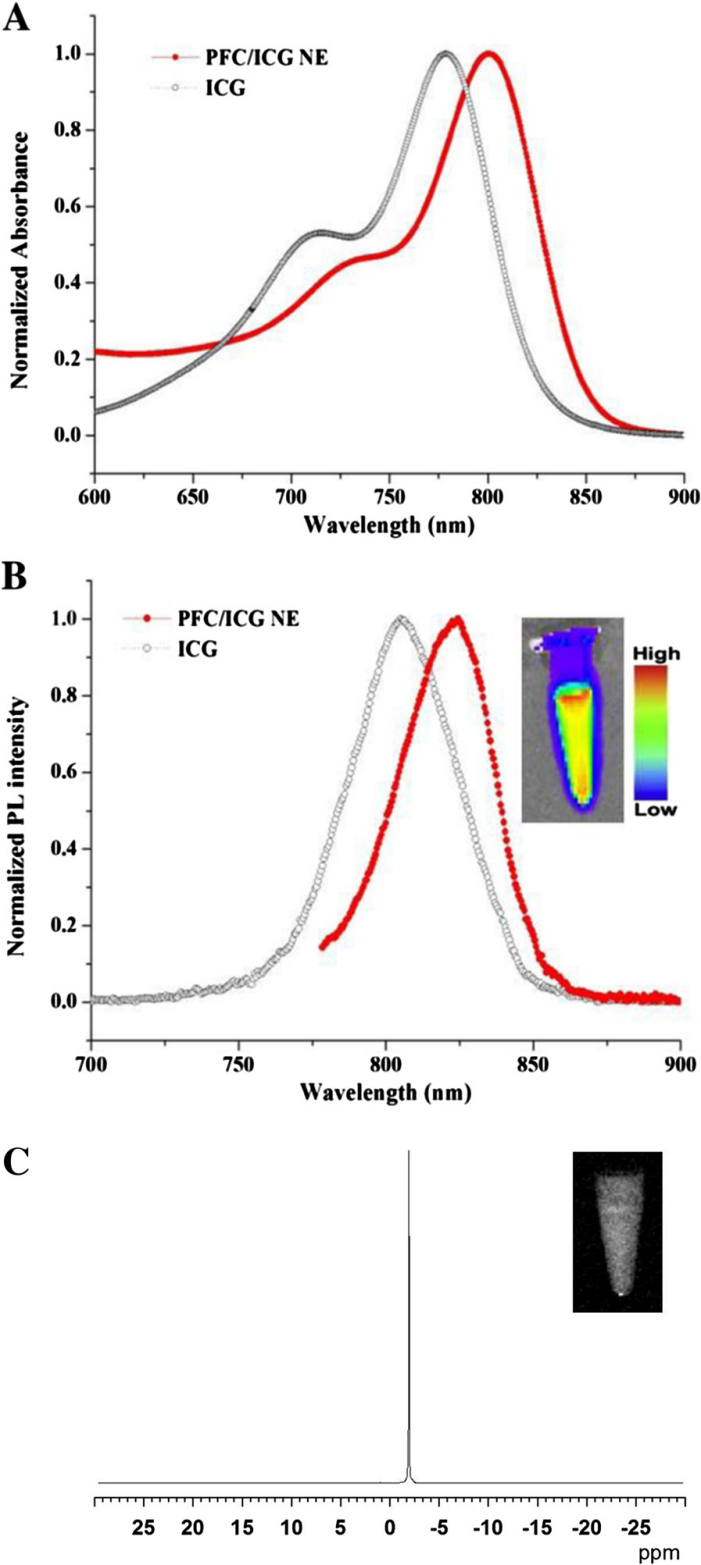
**Characteristics of the PFC/ICG nanoemulsions.** The excitation **(A)** and emission **(B)** spectra of the nanoemulsions. The emission spectra were measured at λex = 760 nm. **(C)** ^19^ F magnetic resonance spectra and imaging of the PFC/ICG nanoemulsion.

To develop suitable MR imaging probes with improved sensitivity for noninvasive in vivo imaging at the cellular and molecular levels, we synthesised multifunctional PFC-based nanoemulsion containing ICG as a NIR organic dye, which provides simultaneous ^19^ F-based MR imaging and NIR optical imaging. ICG has been approved by the FDA for human medical and diagnosis [17]. Also, PFCs were developed for use as a blood substitute [8], and no toxicity, carcinogenicity, or mutagenic effects of PFCs have been reported for pure fluorocarbons [39]. Our synthesis strategy for preparing the multifunctional imaging probe is illustrated in Scheme 1. The bimodal PFC/ICG nanoemulsions were prepared via thin film hydration followed by microfluidisation, as described in the Methods section. The emission spectra of the PFC/ICG nanoemulsion obtained using a fluorescence spectrometer peaked at approximately 825 nm, with a full-width-at-half-maximum (FWHM) of 30 - 35 nm (Figure 1B). Compared with the ICG in water, the absorption and emission spectra of the PFC/ICG nanoemulsions were bathochromically shifted by approximately 20 - 30 nm (Figure 1). These shifts of the PFC/ICG nanoemulsions correspond to the changes in the physicochemical environment and are consistent with a specific interaction of the ICG molecules [16,30]. The red shift toward longer wavelengths resulted in a dramatic decrease of in vivo background signal during detection, leading to an improved signal-to-noise ratio in vivo [22]. The interaction of the ICG molecules within the PFC/ICG nanoemulsions leads to a chemical stabilization of the ICG molecules and can reduce the formation of aggregates [32]. The ^19^ F-based MR spectroscopy data for the PFC/ICG nanoemulsions exhibited a singlet peak (at approximately 36.3 ppm) (Figure 1C). This peak was chosen as the excitation frequency for the PFC/ICG nanoemulsion ^19^ F-MR imaging. The average size and zeta potential of PFC/ICG nanoemulsions were measured using dynamic light scattering analysis (Table 1). The mean diameter of the PFC/ICG nanoemulsions was 119.1 ± 25.1 nm with a polydispersity index of 0.07, and the zeta potential analysis revealed a surface charge of -15.3 ± 0.58 mV (Table 1). The loading efficiency of ICG onto the PFC/ICG nanoemulsions was approximately 95.1 ± 2.2% (Table 1). For comparison, ICG liposomes without a PFC solution had a loading efficiency of 20 ± 3.5%. Encapsulation within the PFC/ICG nanoemulsions resulted in an approximately 4.7-fold increase. The ICG was almost completely encapsulated within the PFC-based nanoemulsion. The PFC nanoparticles core material in PFC/ICG nanoemulsions is surrounded by a lipid monolayer, because these molecules have the property of being both lipophobic and hydrophobic. The ICG molecules can be carried in the PFC-based nanoemulsions for NIR optical imaging. The ICG molecules within these nanoemulsions could successfully lead to the chemical stabilization of the ICG molecules in aqueous solution because of the lipophobic/hydrophobic properties of the PFC molecules.

**Table 1:** **Summary of the physicochemical properties and cytotoxicity activity of the PFC/ICG nanoemulsions**

**Sample**	**Emission peak (nm)**	**Diameter (nm)**	**Polydispersity**	**Loading efficiency (%)**	**CC**_**80**_ ** (μl/ml)** ^**[a]**^		
					**DC2.4**	**HeLa**	**Raw264.7**
PFC/ICG nanoemulsions	825	119.1 ± 25.1	0.07	95.1 ± 2.2	> 100	> 100	> 100
ICG liposome	825	63.4 ± 15.6	0.09	20 ± 3.5	> 100	> 100	> 100

[a] cytotoxicity activity.

### Physicochemical stability of PFC/ICG nanoemulsions

**Figure 2:**
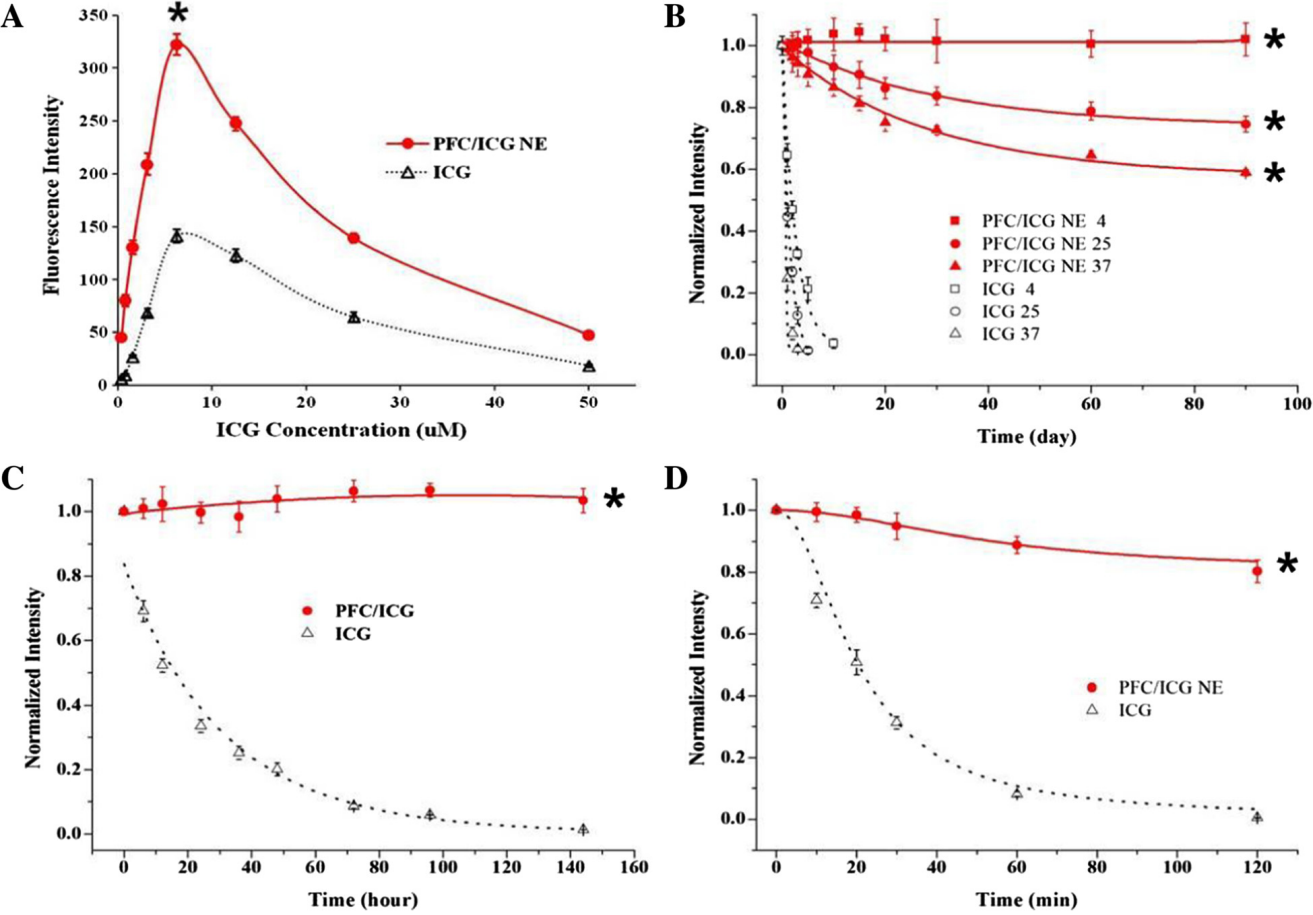
**Physicochemical properties of PFC/ICG nanoemulsions.** **(A)** Plot of fluorescence intensity versus the ICG concentration in the PFC/ICG nanoemulsions or aqueous free ICG. Influence of temperature **(B)**, visible light exposure **(C)**, and NIR lamp **(D)** on the ICG degradation in the PFC/ICG nanoemulsions or ICG solution. The fluorescence intensity of each sample was measured using a spectrofluorometer with excitation and emission wavelengths of 760 nm and 820 nm, respectively. (mean ± SD, *n* = 6). *P < 0.001 compared with the free ICG aqueous.

To investigate the influence of the ICG concentration on fluorescence in aqueous solutions, the fluorescence intensity of PFC/ICG nanoemulsions was measured using ICG concentrations ranging from 0.4 to 50 μM and comparing them to that of free ICG dissolved in water (Figure 2A). The fluorescence intensity increased with increasing ICG concentration up to a maximum of 6.25 μM. Further increase in the ICG concentration causes a gradual decrease in the fluorescence intensity. The PFC/ICG nanoemulsions emitted a 2.3-fold higher fluorescence intensity than the free-ICG solution. This is due to the increased aggregation and self-quenching of free-ICG molecules at high concentration [29]. However, The ICG in PFC/ICG nanomulsions maintains the chemical stabilization and reduces the ICG molecular aggregates. The influence of the external environment on the ICG degradation in the PFC/ICG nanoemulsions was also investigated. The thermal stability of the PFC/ICG nanoemulsions in the dark was determined at 4, 25, and 37°C over 3 months (Figure 2B). Little decrease was noted in the fluorescence intensity of the PFC/ICG nanoemulsions stored at 4°C over the 4-month period (Additional file 1: Figure S2). However, increasing in temperature significantly enhanced the rate of ICG degradation in the PFC/ICG nanoemulsions stored at 25 and 37°C. The fluorescence intensities observed in the PFC/ICG nanoemulsions at 25 and 37°C were approximately 74.6% and 58.9%, respectively. The fluorescence intensity of free-ICG solutions stored at 4, 25, and 37°C disappeared completely within 10 days. To examine whether the encapsulation of the ICG within the PFC/ICG nanoemulsions could enhance the photostability of the ICG fluorescence against external light, the PFC/ICG nanoemulsions were exposed to a NIR LED light at 760 nm or visible light for a predetermined period, and the fluorescence intensity was analyzed. The PFC/ICG nanoemulsions exposed to the visible ambient light maintained their fluorescence intensity over the 6-day period. However, after 1 day of exposure to visible ambient light, the fluorescence intensity of the free-ICG solution was reduced to less than 50% of its initial value and was 3 times less than that of the PFC/ICG nanoemulsions. The exposure to visible light did not affect the fluorescence of the PFC/ICG nanoemulsions. Figure 2D demonstrates the influence of exposure to NIR light on the fluorescence intensity of the PFC/ICG nanoemulsions. After 1 hour of exposure to NIR light, the fluorescence emitted by the PFC/ICG nanoemulsions exhibited an approximately 10.9-fold increase over that of the free-ICG solutions. At the endpoint, the fluorescence of the PFC/ICG nanoemulsions decreased slightly approximately 20%, but the fluorescence of the free-ICG solution completely disappeared. This result suggests that encapsulation of the ICG within PFC/ICG nanoemulsions could successfully protect the ICG fluorescence signals against external environments. The degradation of the ICG with exposure to NIR or visible light is due to the production of photo-excited ICG molecules [40]. The ICG molecules in the PFC/ICG nanoemulsions could efficiently prevent them from aggregating, thus decreasing the fluorescence self-quenching. Therefore, these nanoemulsions could significantly increase the stability and the fluorescence intensity of the ICG and improve its physicochemical stability against external light and temperature changes.

### In vitro fluorescence and ^19^ F-MR imaging

**Figure 3:**
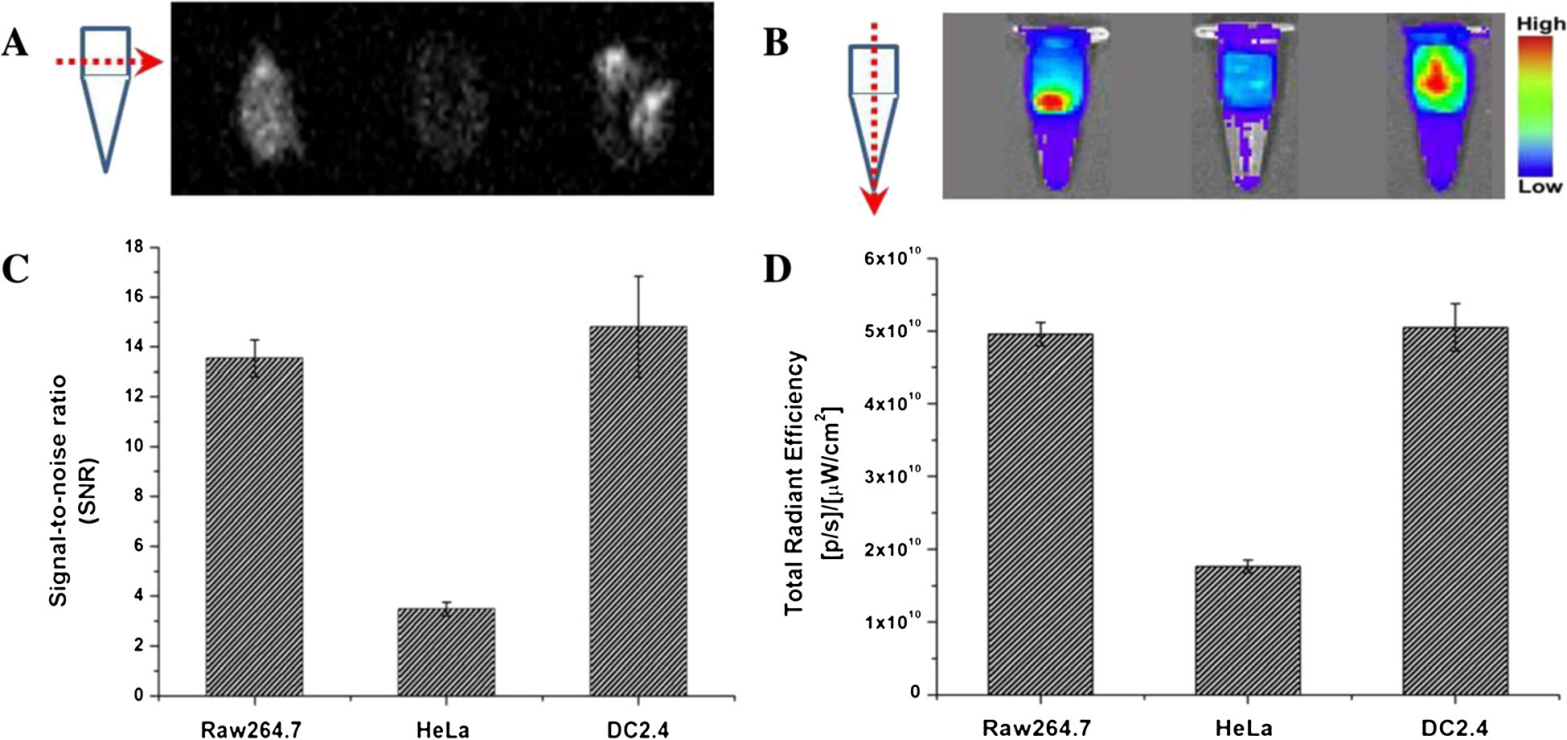
**In vitro** ^**19**^ **F-MR and NIR fluorescence imaging.** ^19^ F-MR images **(A)** and NIR fluorescence images **(B)** of three cell lines (Raw264.7, HeLa, and DC2.4) after incubation with the PFC/ICG nanoemulsions. **(C)** Signal-to-noise ratio for in vitro ^19^ F-MR images in Figure 3A. **(D)** The quantitative analysis of fluorescence intensity in Figure 3B.

**Figure 4:**
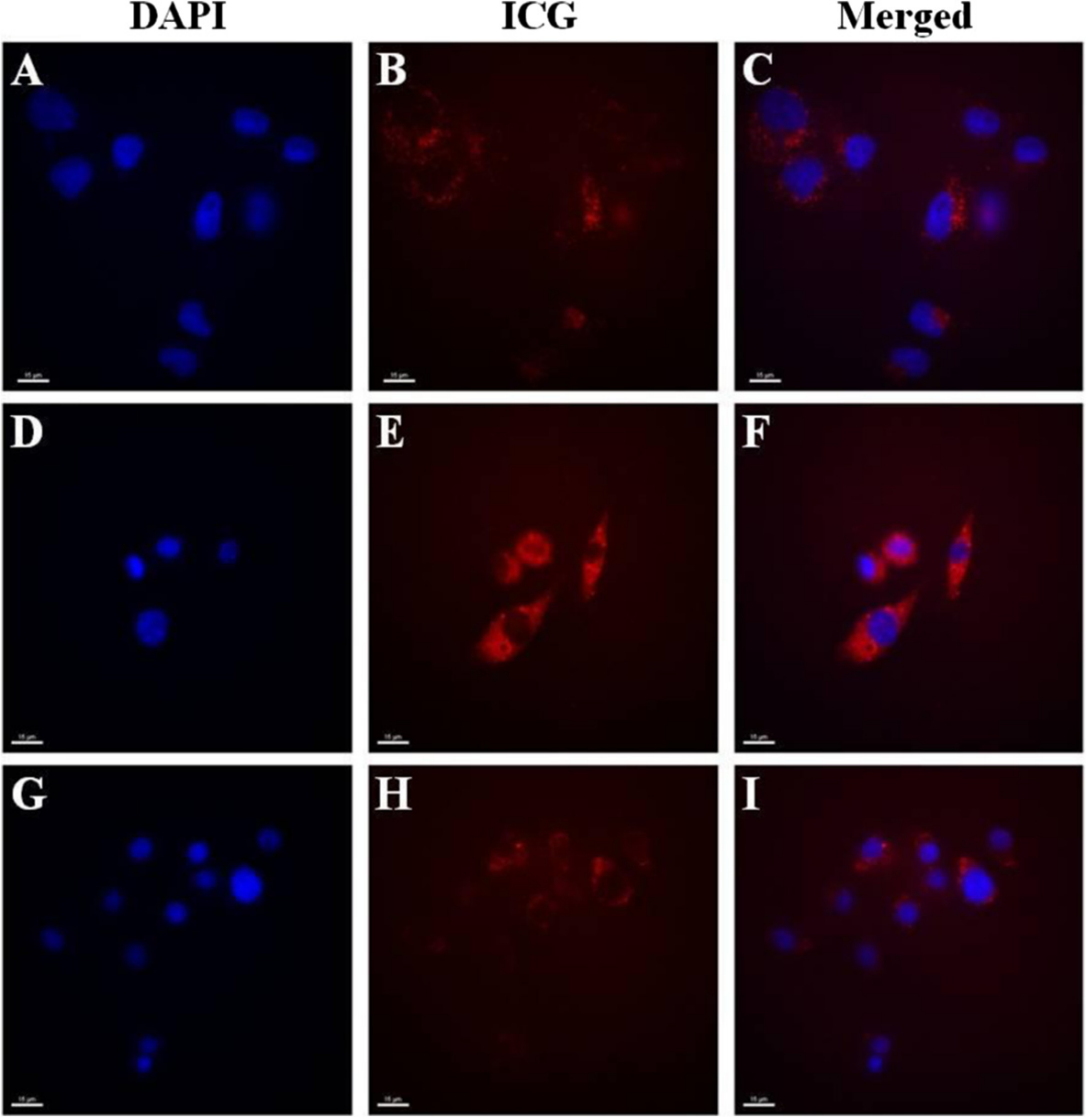
**Cell fluorescence imaging with the PFC/ICG nanoemulsions.** Fluorescence images of HeLa **(A to C)**, DC2.4 **(D to F)**, and Raw264.7 **(G to I)** cells after incubation with the PFC/ICG nanoemulsions. The PFC/ICG nanoemulsions are red, and the DAPI-stained nuclei are blue. Bar, 15 μm.

NIR fluorescence and ^19^ F-MR imaging were performed on PFC/ICG nanoemulsions containing ICG and PFC using IVIS Lumina imaging system (Caliper Life Science, MA) and 4.7 T MR scanner (Biospec, Rheinstetten, Germany), respectively. Figure 3A and B shows the ^19^ F-MR and NIR fluorescence images of three cell lines (Raw264.7, HeLa, and DC2.4) loaded with the PFC/ICG nanoemulsions. The three cell lines were incubated with the PFC/ICG nanoemulsions for 6 h at 37°C, and the NIR fluorescence and ^19^ F-MR images were obtained using IVIS Lumina imaging system and 4.7 T MR scanner, respectively. The NIR fluorescence signals and ^19^ F-MR signals were observed for the three pelleted cell lines. We performed the regions of interest (ROI) analysis to confirm the ^19^ F-MR and NIR fluorescence signals and observed the variations in intensity among the cells (Figure 3C and 3D). These data indicated that the PFC/ICG nanoemulsions could efficiently monitor the intracellular uptake in two phagocytic cell lines (Raw264.7 and DC2.4) suggesting that the PFC/ICG nanoemulsions accumulated in the lymph nodes because they were phagocytosed by phagocytic cells. To evaluate the cell cytotoxicity of the PFC/ICG nanoemulsion, three cell lines were exposed to various concentrations of the PFC/ICG nanoemulsion ranging from 0 to 100 μl/ml for 24 h or 48 h and were evaluated using an MTT assay (Additional file 1: Figure S3). The data revealed that the labeling of the three cell lines with the PFC/ICG nanoemulsion was nontoxic over a broad concentration range, with a CC_80_ of >100 μl/ml (Table 1), indicating that these nanoemulsions are suitable for in vivo animal studies, clinical applications, and biological applications. To determine the intracellular localization of the PFC/ICG nanoemulsions, three cell lines were incubated with the PFC/ICG nanoemulsions for 6 h at 37°C and then stained with a DAPI solution to observe the nuclei. The NIR signals inside the cell membrane exhibited the intracellular uptake of the PFC/ICG nanoemulsions (Figure 4).

### Lymph node mapping

**Figure 5:**
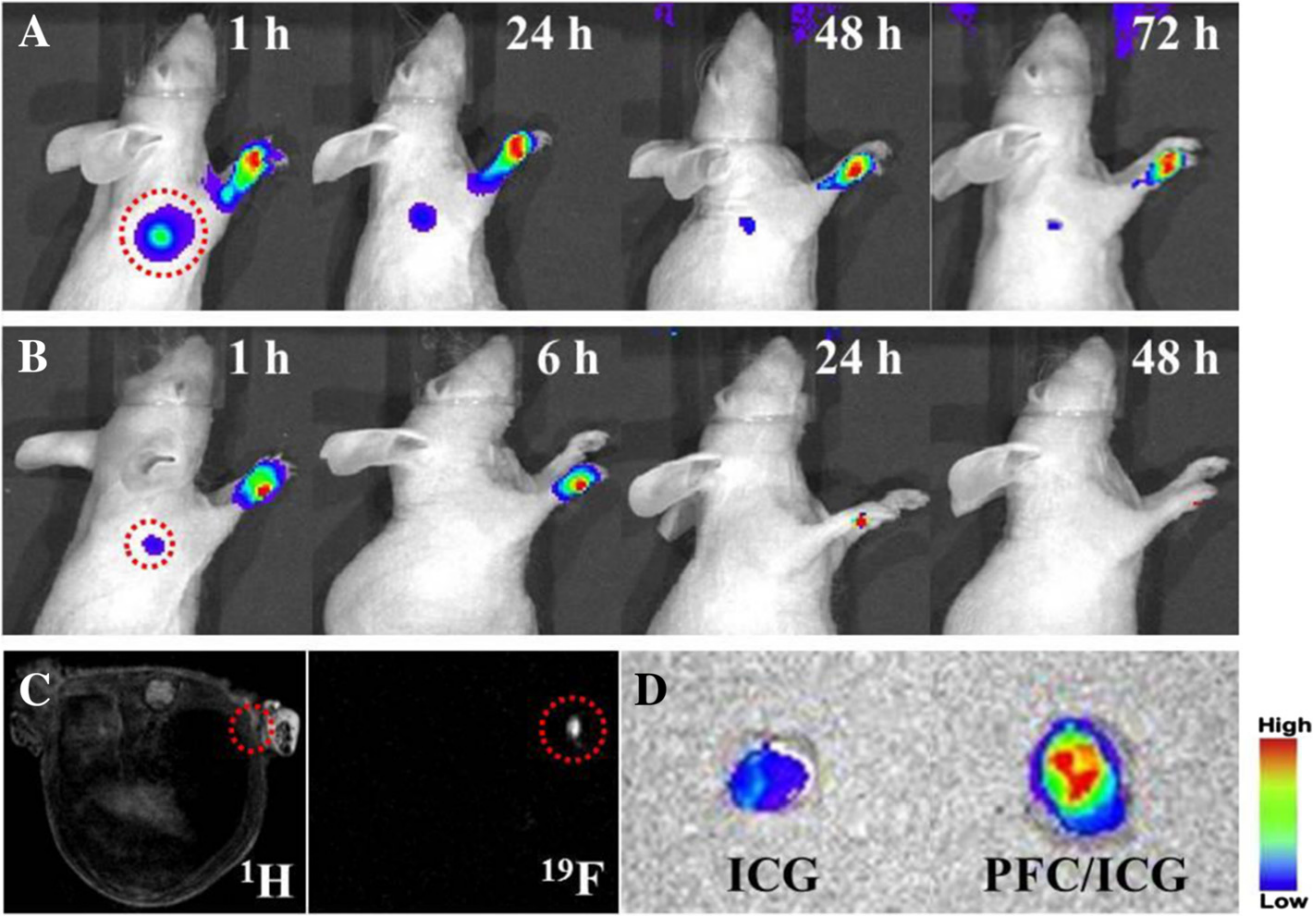
**In vivo NIR image and** ^**19**^ **F-MR image of the sentinel lymph nodes.** NIR fluorescence images at the indicated time following intradermal injection of PFC/ICG nanoemulsions **(A)** and free-ICG solution **(B)** into the right paw of a mouse. **(C)** ^1^H-MR (left) and ^19^ F-MR (right) image following intradermal injection of PFC/ICG nanoemulsions into right paw of a mouse. **(D)** Ex vivo NIR fluorescence images of dissected lymph node at 1 h post-injection.

The use of PFC/ICG nanoemulsion-based bimodal imaging contrast agents was investigated for lymph-node mapping using an IVIS Lumina imaging system and a 4.7 T MRI scanner. For the NIR fluorescence imaging and the in vivo ^19^ F-MR imaging of sentinel lymph nodes, the PFC/ICG nanoemulsions (20 μl of 25 μM) were injected into the footpad of the foreleg of hairless mice. As shown in Figure 5, the ICG NIR signal and the PFC ^19^ F-MR signal were observed in the lymph nodes (red circles). Ex vivo NIR images were obtained of the lymph nodes dissected from the PFC/ICG nanoemulsions-injected mice (Figure 5D), confirming the in vivo imaging results. We performed the ROI analysis to confirm the NIR fluorescence signals (Additional file 1: Figure S4). The NIR fluorescence signal of the PFC/ICG nanoemulsions in the lymph nodes was significantly prolonged compared with that of the free-ICG solution. The PFC/ICG nanoemulsions presented significant NIR fluorescence signals even at 72 h post-injection. In contrast, no detectable signal was recorded in the lymph nodes from the free-ICG solution at 6 h post-injection. The relatively short in vivo fluorescence of the free-ICG is attributed to the fluorescence quenching of the free-ICG in physiological environments and its rapid aggregation and clearance from the body [16,30,41]. Because ICG is amphiphilic properties, it can bind to lipoprotein and plasmatic proteins. This binding leads to its removal by hepatic parenchymal cells and its secreted into the bile [41]. However, the ICG molecules encapsulated inside the PFC/ICG nanoemulsions were protected from the physicochemical environment, suggesting that the lymph node mapping could be tracked effectively using PFC/ICG nanoemulsions as bimodal imaging contrast agents.

## Conclusion

In summary, we encapsulated ICG molecules inside the PFC/ICG nanoemulsions as a novel bimodal imaging probe to allow for simultaneous ^19^ F-MR imaging and NIR optical imaging. The conjugation chemistry of ICG molecules is difficult due to their amphiphilicity and few functional groups. However, ICG molecules were encapsulated through a simple method to improve their properties. The ICG molecules protected the PFC/ICG nanoemulsions from aggregation and thus decreased the fluorescence self-quench. Therefore, these nanoemulsions could significantly increase the in vivo and in vitro stability and fluorescence intensity of ICG and improve its physicochemical stability against external light and temperature. Also, we used multi-modal PFC/ICG nanoemulsions to indentify the sentinel lymph nodes. Lymph nodes were detected by NIR optical imaging and ^19^ F-MR imaging. For sentinel lymph node biopsy, the incision procedure of sentinel nodes should be performed within short time (~30 min) because of the easy diffusion of free ICG and the decrease of fluorescence signal. The accuracy of sentinel lymph node biopsy depends upon the detection of sentinel nodes with high sensitivity and long-lasting vital dye. This result showed the suitability of the proposed nanoemulsions for noninvasive lymph node mapping as they enable long-time detection of lymph nodes. In the future, molecular probes in combination with various imaging modalities will provide more effective image-guided therapeutic tools for diagnostics, prognostics, and the treatment of diseases in diverse clinical settings.

## Additional file

Additional file 1: Figure S1. TEM image of PFC/ICG nanoemulsion. **Figure S2.** Fluorescence intensity of the PFC/ICG nanoemulsions or free ICG at varying incubation times. Samples were excited at 760 nm. The emission spectra of the PFC/ICG nanoemulsions and free ICG solutions were collected at 825 nm or 805 nm, respectively. (mean ± SD, *n* = 6). **Figure S3.** In vitro cytotoxicity of the PFC/ICG nanoemulsions in HeLa, DC2.4, and Raw264.7 cells. The cells were incubated with the PFC/ICG nanoemulsion for 24 and 48 h at 37°C and the viability of the cells were evaluated with increasing concentrations of the nanoemulsions ranging from 0.41 to 100 μL mL^-1^ using an MTT assay (mean ± SD, *n* = 6). **Figure S4.** The quantitative analysis of the fluorescence intensity after intradermal injection of either the PFC/ICG nanoemulsions or free-ICG solution into the foodpad of the foreleg. A) foot, B) Lymph node.
