# Supplementary material for: Highly enhanced optical properties of indocyanine green/perfluorocarbon nanoemulsions for efficient lymph node mapping using near-infrared and magnetic resonance imaging
Source: Nano Converg. 2014 Mar 14;1(1):6. doi: 10.1186/s40580-014-0006-6 (PMC5271138; doi:10.1186/s40580-014-0006-6)
Supplement: Additional file 1: Figure S1. — TEM image of PFC/ICG nanoemulsion. Figure S2. Fluorescence intensity of the PFC/ICG nanoemulsions or free ICG at varying incubation times. Samples were excited at 760 nm. The emission spectra of the PFC/ICG nanoemulsions and free ICG solutions were collected at 825 nm or 805 nm, respectively. (mean ± SD, n = 6). Figure S3. In vitro cytotoxicity of the PFC/ICG nanoemulsions in HeLa, DC2.4, and Raw264.7 cells. The cells were incubated with the PFC/ICG nanoemulsion for 24 and 48 h at 37°C and the viability of the cells were evaluated with increasing concentrations of the nanoemulsions ranging from 0.41 to 100 μL mL-1 using an MTT assay (mean ± SD, n = 6). Figure S4. The quantitative analysis of the fluorescence intensity after intradermal injection of either the PFC/ICG nanoemulsions or free-ICG solution into the foodpad of the foreleg. A) foot, B) Lymph node. [file 40580_2014_6_MOESM1_ESM.pdf]

Supporting Information

Highly enhanced optical properties of indocyanine green/perfluorocarbon nanoemulsions for efficient lymph node mapping using near-infrared and magnetic resonance imaging

Pan Kee Bae, Juyeon Jung, Bong Hyun Chung*

Bionanotechnology Research Center, Korea Research Institute of Bioscience and Biotechnology, Daejeon 305-806, Korea

* CORRESPONDING AUTHOR EMAIL ADDRESS: chungbh@kribb.re.kr

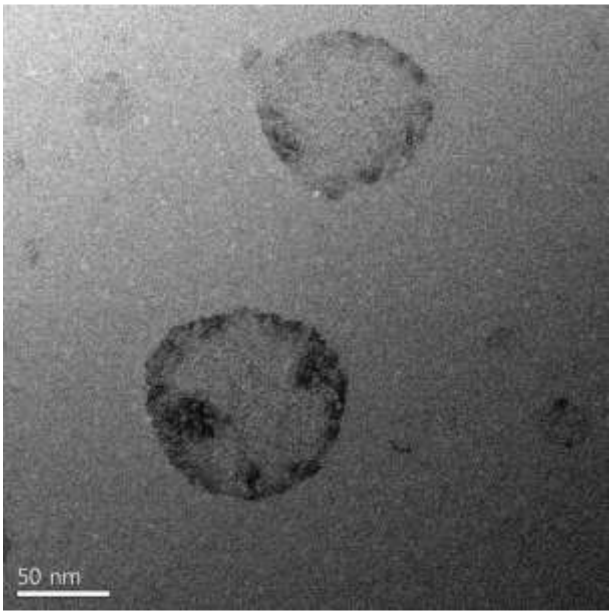

Figure S1. TEM image of PFC/ICG nanoemulsion.

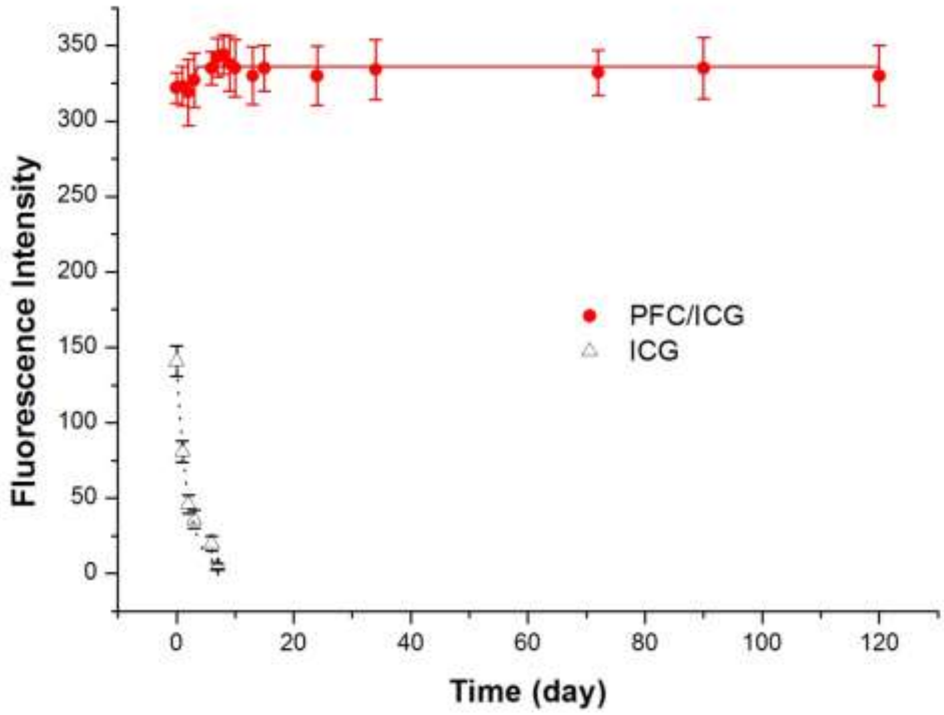

Figure S2. Fluorescence intensity of the PFC/ICG nanoemulsions or free ICG at varying incubation times. Samples were excited at 760 nm. The emission spectra of the PFC/ICG nanoemulsions and free ICG solutions were collected at 825 nm or 805 nm, respectively. (mean ± SD, $n = 6$)

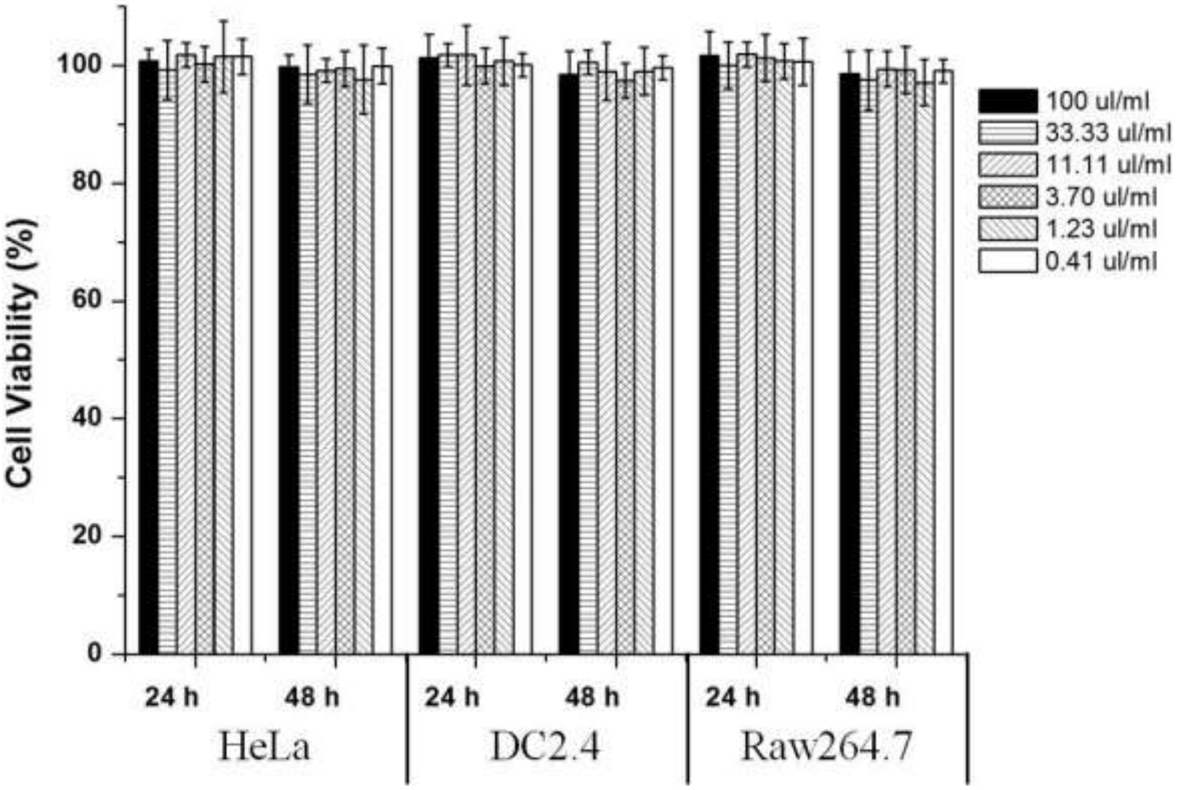

Figure S3. *In vitro* cytotoxicity of the PFC/ICG nanoemulsions in HeLa, DC2.4, and Raw264.7 cells. The cells were incubated with the PFC/ICG nanoemulsion for 24 and 48 h at 37°C and the viability of the cells were evaluated with increasing concentrations of the nanoemulsions ranging from 0.41 to 100 μL mL^{-1} using an MTT assay (mean ± SD, *n* = 6).

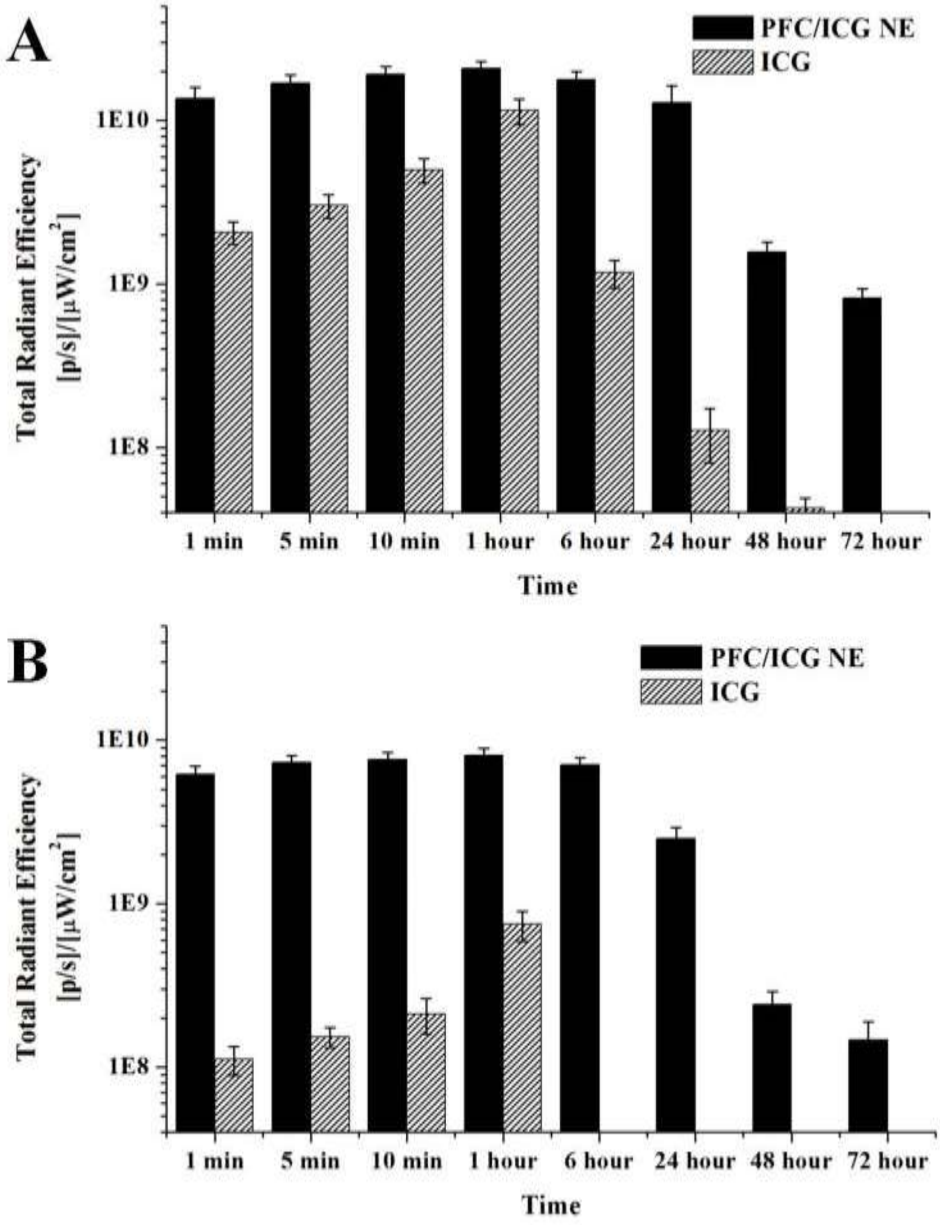

Figure S4. The quantitative analysis of the fluorescence intensity after intradermal injection of either the PFC/ICG nanoemulsions or free-ICG solution into the foodpad of the foreleg. A) foot, B) Lymph node.
